# Benzodiazepines and Related Drugs as a Risk Factor in Alzheimer's Disease Dementia

**DOI:** 10.3389/fnagi.2019.00344

**Published:** 2020-01-08

**Authors:** Miren Ettcheto, Jordi Olloquequi, Elena Sánchez-López, Oriol Busquets, Amanda Cano, Patricia Regina Manzine, Carlos Beas-Zarate, Rubén D. Castro-Torres, Maria Luisa García, Mónica Bulló, Carme Auladell, Jaume Folch, Antonio Camins

**Affiliations:** ^1^Departament de Farmacologia, Toxicologia i Química Terapèutica, Facultat de Farmàcia i Ciències de l'Alimentació, Universitat de Barcelona, Barcelona, Spain; ^2^Departament de Bioquímica i Biotecnologia, Facultat de Medicina i Ciències de la Salut, Universitat Rovira i Virgili, Reus, Spain; ^3^Institut de Neurociències, Universitat de Barcelona, Barcelona, Spain; ^4^Biomedical Research Networking Centre in Neurodegenerative Diseases (CIBERNED), Madrid, Spain; ^5^Laboratory of Cellular and Molecular Pathology, Facultad de Ciencias de la Salud, Instituto de Ciencias Biomédicas, Universidad Autónoma de Chile, Talca, Chile; ^6^Unitat de Farmàcia, Tecnologia Farmacèutica i Fisico-química, Facultat de Farmàcia i Ciències de l'Alimentació, Universitat de Barcelona, Barcelona, Spain; ^7^Institute of Nanoscience and Nanotechnology (IN2UB), Universitat de Barcelona, Barcelona, Spain; ^8^Department of Gerontology, Federal University of São Carlos (UFSCar), São Carlos, Brazil; ^9^Laboratorio de Regeneración y Desarrollo Neural, Departamento de Biología Celular y Molecular, Instituto de Neurobiología, CUCBA, Guadalajara, Mexico; ^10^Institut d'Investigació Sanitària Pere Virgili (IISPV), Reus, Spain; ^11^Centro de Investigación Biomédica en Red Fisiopatologia de la Obesidad y la Nutrición (CIBEROBN), Institut de Salud Carlos III, Madrid, Spain; ^12^Departament de Biologia Cellular, Fisiologia i Immunologia, Facultat de Biologia, Universitat de Barcelona, Barcelona, Spain

**Keywords:** benzodiazepines, Alzheimer's disease, dementia, cognition, risk factors

## Abstract

Benzodiazepines (BZDs) and Z-drugs are compounds widely prescribed in medical practice due to their anxiolytic, hypnotic, and muscle relaxant properties. Yet, their chronic use is associated with cases of abuse, dependence, and relapse in many patients. Furthermore, elderly people are susceptible to alterations in pharmacodynamics, pharmacokinetics as well as to drug interaction due to polypharmacy. These situations increase the risk for the appearance of cognitive affectations and the development of pathologies like Alzheimer's disease (AD). In the present work, there is a summary of some clinical studies that have evaluated the effect of BZDs and Z-drugs in the adult population with and without AD, focusing on the relationship between their use and the loss of cognitive function. Additionally, there is an assessment of preclinical studies focused on finding molecular proof on the pathways by which these drugs could be involved in AD pathogenesis. Moreover, available data (1990–2019) on BZD and Z-drug use among elderly patients, with and without AD, was compiled in this work. Finally, the relationship between the use of BZD and Z-drugs for the treatment of insomnia and the appearance of AD biomarkers was analyzed. Results pointed to a vicious circle that would worsen the condition of patients over time. Likewise, it put into relevance the need for close monitoring of those patients using BZDs that also suffer from AD. Consequently, future studies should focus on optimizing strategies for insomnia treatment in the elderly by using other substances like melatonin agonists, which is described to have a much more significant safety profile.

## Introduction

**Figure 1:**
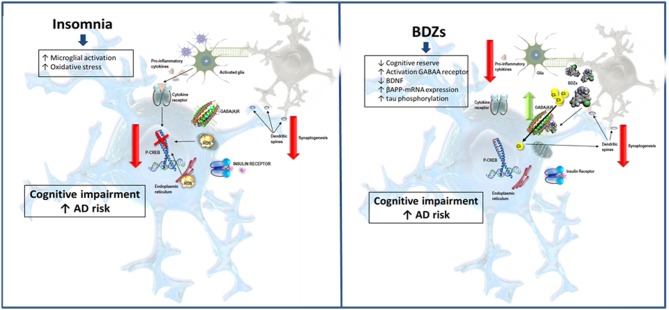
Schematic representation of potential pathways by which insomnia and benzodiazepines could increase AD risk. Insomnia is a CNS stressor, which induces microglial activation and oxidative stress. Likewise, oxidative stress may be involved in cognitive impairment by decreasing phosphorylation levels of p-CREB and altering dendritic spines and synapses. Moreover, sleep disturbances prevent clearing toxic metabolites such as β-amyloid. These lead to an increased production of inflammatory cytokines and the formation of Aβ-plaques. In turn, BZDs activate GABA_A_ receptors, thus interfering excitatory synapses and decreasing cognitive reserve. Moreover, these drugs have been shown to decrease BDNF as well as increase β-amyloid precursor protein (APP) mRNA levels and tau phosphorylation. All these mechanisms could increase the risk of cognitive impairment through neuroinflammation, decrease synaptic plasticity and brain insulin signaling as well as accumulation of Aβ plaques and neurofibrillary tangles.

Sleep disturbances have been reported to increase Amyloid Beta (Aβ) levels in the cerebrospinal fluid of healthy subjects, contributing to the advancement of neurodegeneration and the appearance of mild cognitive impairment (MCI) (Lopez et al., 1999; Virta et al., 2007; Modabbernia et al., 2011; Consensus and Statements, 2014; Di Meco et al., 2014; Benedict et al., 2015; Gage et al., 2015; Chen et al., 2016; Gaugler et al., 2016; Kincheski et al., 2017; La Frenais et al., 2017; Livingston et al., 2017; Atkin et al., 2018; Burke et al., 2018). At preclinical level, it has been described that sleep deprivation in 3xTg mice acts as a chronic stressor, favoring the decrease of Cyclic adenosine monophosphate (cAMP) response element binding (CREB) and affecting synaptic plasticity and cognitive functions (Di Meco et al., 2014) (Figure 1). It has been described that sleep restriction increases susceptibility to Amyloid beta (Aβ)-induced memory impairment in mice (Kincheski et al., 2017), accompanied by increased plasma levels of corticosterone, just like higher levels of brain pro-inflammatory cytokines [tumor necrosis factor alfa (TNFα), interleukin 1 beta (IL1-β) and IL-6], which contributed to memory impairment and synapse damage. Consequently, sleep alterations have become major risk factors for the development of sporadic pathologies like AD and need to be properly managed by drugs that will restore balanced physiological sleep periods (Kincheski et al., 2017; Hennawy et al., 2019).

Benzodiazepines (BZDs) and their analogous Z-drugs are psychotropic drugs commonly used in medical practice against anxiety, nervousness, convulsive states, depression, and psychosis. They also act as skeletal muscle relaxants and hypnotics for the treatment of short-term acute insomnia (Dolder et al., 2007). On a molecular level, BZDs and Z-drugs facilitate the inhibitory activity of the neurotransmitter gamma-aminobutyric acid (GABA) on its receptor (Duke et al., 2018), favoring the flow of chlorine ions through the ionotropic channel bound to the receptor and producing the hyperpolarization of neuronal membranes (Sigel and Ernst, 2003). The GABA_A_ receptor is an ionotropic receptor composed of five protein subunits that mediate different behavioral and pharmacological responses (Mehdi, 2012; Duke et al., 2018). The α1 subunit of the GABA_A_ receptor is thought to be responsible for sedative effects, while the α2 and α3 subunits exert anxiolytic and antidepressant activities. Finally, the α5 subunit is involved in the control of cognitive functions such as memory and learning (Rissman et al., 2007; Savić et al., 2010).

From a pharmacokinetic point of view, BZDs and related drugs are divided into three groups according to their half-life. It can either be long (over 24 h), intermediate (between 6 and 24 h), or short (<6 h). Usually, short and intermediate-acting BZDs are prescribed for insomnia, while longer-acting BZDs are reserved for anxiety, but their effects can vary with age and liver metabolic capacity. Old age is associated with a decrease of oxidative metabolism, causing an extension on drug half-life due to changes in pharmacokinetics and pharmacodynamics (Taipale et al., 2015; Hessmann et al., 2018). In fact, the prolonged use of these drugs (over 2 months) in advanced age has shown to produce serious side effects, causing tolerance and dependence, increased risk of falls and fractures as well as an impairment of cognitive processes (Pharmd et al., 2003; Obradovi et al., 2005; Stewart, 2005; Rissman et al., 2007; Savić et al., 2010; Rosenberg et al., 2012; Makaron et al., 2013; Biétry et al., 2017; Nørgaard et al., 2017; Duke et al., 2018; Picton and Pharm, 2018; Underlien et al., 2018; Scott and Aricescu, 2019).

The effects of BZDs and other hypnotic drugs on cognition in elderly patients are intensive areas of research nowadays (Carlisle, 2017). In a recent study, Kurlawala et al. reported a case of a 76-year-old male who presented onset of short-term memory loss after a 3 year treatment with a BZD (Kurlawala et al., 2018). Magnetic resonance imaging studies revealed that cognitive loss could be a result of atrophy in the hippocampus and cortex (Barker et al., 2004; Hessmann et al., 2018; Kurlawala et al., 2018; Picton and Pharm, 2018). Studies performed by the groups of Glass et al. and Kripke et al. reported that the adverse effects of hypnotics outweigh the benefit they achieve in the population older than 60 years, going so far as to increase mortality risk in some patients (Glass et al., 2005; Kripke et al., 2012; Hammond and Esclapez, 2015). Consequently, these results have led the National Institutes of Health and the Beers Criteria of the American Geriatric Society to list molecules, such as eszopiclone, zolpidem, and zaleplon, as “potentially inappropriate medications” (Consensus and Statements, 2014; Investigations, 2015; Taipale et al., 2015; Wennberg et al., 2018). It was determined that BZDs should be avoided or should only be prescribed for short and specific situations (Letter, 2019; Walsh and Roth). However, BZDs and Z-drugs are still widely inappropriately prescribed in sleepless patients (Gunja, 2013; Pariente et al., 2016; Nørgaard et al., 2017; Richardson et al., 2018).

Luckily, alternatives to BDZs exist to treat situations of sleep deprivation. Sateia et al. published a clinical practice guideline for the treatment of insomnia in the Journal of Clinical Sleep Medicine (Sateia et al., 2017a,b). In their manuscript, the authors suggest ramelteon for sleep-onset insomnia. This drug selectively binds to the melatonin receptors, avoiding dependence and other important side effects associated with BZD long-term treatment. In the same paper, the authors also recommend Z-drugs or BZD hypnotics for sleep maintenance in insomnia (Sateia et al., 2017a). In 2018, the U.S. Food and Drug Administration (FDA) approved several non-BZD compounds for the treatment of insomnia (Richardson et al., 2018). These include antidepressants with anxiolytic or sedative action, such as escitalopram, doxepin, trimipramine, and amitriptyline as well as heterocyclic drugs like trazodone and mirtazapine (Gunja, 2013; Richardson et al., 2018).

The objective of this article was to review and discuss published material on the risk of BZD and Z-drug use and their role in the appearance of cognitive loss in cases of AD.

## The Potential Molecular Mechanisms Involved in Benzodiazepines and Related Drug Induced Cognitive Impairment

Although the molecular mechanisms by which BZDs and psychotropic drugs could induce cognitive impairment are uncertain, several hypotheses have been suggested (Gage et al., 2015). These mechanisms are summarized in Figure 1.

One hypothesis states that elderly long-term consumers of BZDs show limited cognitive reserve capacity. This concept refers to the ability to tolerate age-related and disease-related changes in the brain due to the existence of strong and redundant synaptic connections in the brain. This mechanism would allow resisting longer neurodegenerative pathologies without developing clear cognitive clinical symptoms or memory loss (Stern, 2012). Since BZDs and Z-drugs are positive GABA_A_ receptor modulators, they decrease brain activation and reduce synaptic plasticity, affecting the patient's ability to create a new memory. Thus, BZDs interfere with the function of excitatory synapses, which is required for memory. In addition, the loss of social networks in aging people could act as an additional factor that may also affect cognitive function. Likewise, BZD treatment for sleep disturbances in aging could limit the capacity to create social communication networks and precipitate the development of dementia (Wan et al., 2004; Pariente et al., 2016; Mohamad et al., 2019).

The composition of GABA_A_ receptors could also be involved in cognitive alterations related to hypnotic drugs. It has been reported that the binding of BZDs to the α5GABA_A_ subunit, which is almost exclusively found in the hippocampus and deep layers of the cortex, impairs memory for contextual information in monkeys (Wan et al., 2004; Mohamad et al., 2019). Of note, Zolpidem did not impair the performance of a task based on visual cues which could be explained by its affinity for the α1GABA_A_, instead of α5GABA_A_ (Mohamad et al., 2019). Moreover, it has been reported that the memory-impairing effects of BZDs are not blocked by the α1GABAA-selective BZD antagonist β-carboline-3-carboxylate-3-butyl-ester, whereas the α5GABA_A_ antagonist XLi-093 blocked the effects of triazolam but not zolpidem (Caraiscos et al., 2004; Mohamad et al., 2019). These findings suggest a specific role of α5GABA_A_ receptor in BZD-related cognitive impairment. Furthermore, recent reports suggest that the modulation of extrasynaptic tonic inhibition of α5GABAA in the CA1 hippocampus and cerebral cortex could improve and regulate memory processes in the hippocampus. Therefore, negative modulation of α5GABA_A_ could be a suitable target for the development of potential therapies against cognitive dysfunction in neurological diseases (Caraiscos et al., 2004). Contrarily, Joksimović et al. demonstrated that α1GABA_A_ subunit receptor activation may affect the spatial learning performance in rodents (Joksimović et al., 2013). They also reported that α1GABA_A_ subunit is involved in anterograde amnesia, sedation, motor incapacitation, and anticonvulsant BZD effects.

On another front, the “GABAergic deafferentation hypothesis of brain aging” introduced by Marczynski is based on the fact that administration of diazepam to rats causes a diminution of glucose utilization in the brain (Marczynski, 1995, 1998). Since a decrease in cellular adenosine triphosphate (ATP) levels is a feature of the aging process and AD, it would be logical to consider a potential metabolic influence of BZDs–GABA receptor complex in the brain, predisposing to AD in aging (Marczynski, 1995). Regarding this, the administration of flumazemil, an antagonist of BZDs, increases glucose utilization in rodents (Marczynski, 1998). Thus, a potential mechanism explaining the deleterious effects of BZDs on cognitive processes could be the depolarization and the depressive action of BZD agonists leading to brain energy metabolism deficit. In addition, BZD agonists may inhibit the effects of paracrine-autocrine neurotrophin family. Indeed, it is well-known that nerve growth factor (NGF)-related proteins participate in the regulation of survival, growth, and maintenance of neurons. In this sense, Zhao et al. reported that mice under long-term diazepam administration showed behavior alterations and reduced hippocampal synaptophysin and brain-derived neurotrophic factor (BDNF) levels (Zhao et al., 2012). This is a matter of importance since BDNF influences the functional aspects of synaptic transmission by (i) enhancing the number of α-amino-3-hydroxy-5-methyl-4-isoxazolepropionic acid (AMPA) receptors in the postsynaptic membrane, (ii) enhancing Long term potentiation (LTP), and (iii) reducing GABA_A_ receptor expression and decreasing inhibitory GABA-ergic neuro-transmission in the hippocampus (Jovanovic, 2004). Through this mechanism, BZDs could inhibit the axonal transport in both directions, increase the formation of neurofibrillary tangles, and also induce the βAPP-mRNA gene expression, thus increasing the risk of AD. Furthermore, the glutamate levels could also be affected by destabilizing neuronal Ca^2+^ homeostasis, and neurons could be more sensitized to the effects of glutamate (Jovanovic, 2004).

Recently, an interesting study of Whittington and collaborators reported that midazolam increased tau phosphorylation in C57BL/6 mice (Whittington et al., 2019). Consequently, the authors suggested that the effects of the most frequently used BZDs on tau phosphorylation should be evaluated in deep since they could be strongly involved in the increase of AD risk. Indeed, pathogenic forms of tau, including soluble tau oligomers, can promote neuronal dysfunction and cognitive loss by several mechanisms at the early stages of disease (Forner et al., 2017; Tracy and Gan, 2018). Likewise, Marciniak and collaborators reported that tau protein could be involved in the regulation of brain insulin signaling, which plays a fundamental role in the cognitive process (Marciniak et al., 2017). They demonstrated that the alteration of insulin signaling in preclinical models of AD could occur through alterations of tau.

It is also well-known that apolipoprotein E (APOE) 4 allele is a risk factor of AD (Stonnington et al., 2009). The presence of this allele is associated with an increased Aβ accumulation as well as with an increase in cognitive decline and disease development when compared to other APOE allelic variants. In this respect, Pomara and collaborators reported an increased sensitivity to the cognitive adverse effects of acute doses of lorazepam in elderly carriers of the APOE4 allele (Pomara et al., 2011). Thus, it seems that APOE4 could also be a risk for psychotropic drug-mediated cognitive decline. Likewise, the same group suggested that subjects who carry the very long Translocase of Outer Mitochondrial Membrane 40 Homolog (TOMM40) Poly-T Length and do not possess the ϵ4 allele might also be at increased risk for BZD-related cognitive loss. Therefore, the influence of APO E genotype on hypnotics as risk factors for AD seems relevant, and APOE4 genotyping could be useful in guiding physicians about avoiding BZD prescription in at risk patients. Finally, Stonnington and collaborators reported that acute 2 mg dose of lorazepam given to middle aged (50–65 years) cognitive normal adults caused higher decline in verbal episodic memory and visuospatial memory/executive function in ε4 carriers compared to non-carriers (Stonnington et al., 2009).

However, Aβ might also have indirect effects on the inhibitory GABAergic transmission as a result of the dynamic GABAergic balance modulation of the other two excitatory systems (cholinergic and glutamatergic neurotransmission). Interestingly, it has recently been suggested that the imbalance between excitatory and inhibitory systems underlies the synaptic dysfunction caused by Aβ (Rissman et al., 2007).

## Clinical Studies

Between November 2018 and February 2019, we performed a literature review on clinical studies linked to the research topic in three acknowledged databases: Web of Science, Scopus, and PubMed. The collocated keywords were as follows: Alzheimer's disease AND benzodiazepines, Benzodiazepines AND cognitive impairment, Benzodiazepines AND cognitive decline, hypnotics AND cognitive decline, Z-drugs AND cognitive impairment, hypnotics AND Alzheimer's disease. The keywords were combined and integrated in the database and journal searches. The terms used were searched using AND to combine the keywords listed and using OR to remove search duplication where possible. References of retrieved articles that the authors' searches may have missed or could have been ignored were also assessed.

All these studies were fully investigated and considered under the following inclusion criteria:

- All articles had to be published studies conducted on human subjects up to February 2019.
- All articles had to be written in English.
- The primary outcome had to be focused on cognitive decline and Alzheimer's disease.

### Case–Control Studies

Data from studies reviewed in this section can be found in Table 1.

**Table 1:** Overview of selected case–control studies exploring the effect of BZDs and Z-drugs on the delay of cognitive decline in the elderly and Alzheimer's disease patients.

**References**	**Objective**	**Intervention**	**Number of subjects**	**Main outcome measures**	**Findings**
Lagnaoui et al. (2002)	To investigate link between BZD and dementia in a large representative cohort of French community dwelling population. Data from PAQUID (Personnes Agées Quid) Research Program in Bordeaux.	1989–1997	150 cases and 3,519 controls. Age ≥ 65. Data from the UK-based Clinical Practice Research Datalink	Cognitive impairment was evaluated using the Mini- Mental Status Evaluation (MMSE) and CT scanner. Diagnosis was based on Diagnostic and Statistical Manual of Mental Disorders (DSM-III-R) and NINCDS-ADRDA	BZD consumption constitutes a risk factor for dementia in the elderly.
Wu et al. (2009)	To explore the association between long-term BZD use and the risk of dementia. Nested case–control study (Taiwan)	1997–2004	4,626 control subjects, and 779 dementia patients treated with hypnotics. Age ≥ 45.	Cumulative dose DDD of sedative-hypnotics and average days, per year.	Long-term use of hypnotic-sedative drugs increases AD risk.
Wu et al. (2011)	To explore if BZDs discontinuation affects the risk of dementia. Nested case–control study (Taiwan)	1997–2007	8,434 patients with dementia and 16.706 control subjects. Age ≥45.	BZD discontinuation.	The risk of AD increases with BDZs, but it decreases with BZDs discontinuation.
Billioti de Gage et al. (2012)	To evaluate the association between use of BZDs and dementia.	1987–1989	1,063 community dwelling people. Age ≥ 65.	Dementia evaluated based on the Diagnostic and Statistical Manual of Mental Disorders, third edition, revised (DSM-III-R).	The use of BDZs was associated with increased risk of dementia.
Billioti de Gage et al. (2014)	To evaluate the association between former BZD use and the risk of AD and to investigate the potential dose–effect relation (Canada)	2000–2009	1,796 AD patients and 7.184 controls. Age >66.	First diagnosis (index date) of AD (ICD-9 (international classification of disease, ninth revision)	No dose-effect relation between BZDs and increased risk of AD was found in older people treated previously for more than 3 months.
Imfeld et al. (2015)	To assess the association of BZD use with risk of dementia.	1998–2013	16,823 subjects with AD and 9,636 subjects with vascular dementia and each being randomly matched (age, sex, general practice and duration of follow-up) with one control. Age ≥ 65. The time of study with these BZDs was 2 years from the diagnostic of AD and 3 years from vascular dementia	An algorithm based on recordings of specific dementia tests [e.g., Mini-Mental State Examination (MMSE), Clock Drawing Test (CDT), or Abbreviated Mental Test (7-Min Screen)], referrals to specialists, brain imaging [computed tomography (CT), magnetic resonance imaging (MRI), or single photon emission computed tomography (SPECT)] symptoms (memory impairment, aphasia, apraxia, or agnosia) supportive of a diagnosis of a specific dementia subtype.	Long-term BZDs use is not associated with an increased risk of AD or vascular dementia.
Gomm et al. (2016)	To explore the association between BDZ and Z-drug consumption and dementia in a large German population over 60 years old in German public health insurance data Allgemeine Ortskrankenkassen (AOK), which covers about 50% of the population at least 80 years old	2004–2011 follow-up.	21,145 cases (any dementia) and 84,580 controls, over 60 years of age.	Cognitive tests.	Regular use of BDZs and Z-drugs in the elderly induces a significantly increased risk of dementia.
Saarelainen et al. (2016)	The authors evaluated the effect of BZDs and Z-drugs administered 2 years before and three years after the diagnosis of AD. MEDALZ cohort in Finland.	2005–2011	51,981 patients with AD and 159.974 controls.	AD diagnoses based on the National Institute of Neurologic and Communicative Disorders and Stroke and the Alzheimer's disease and Related Disorders Association as well as the Diagnostic and Statistical Manual, Fourth Edition, criteria.	BZD use is higher in AD patients. BDZs could decrease the effectiveness of anti-AD drugs.
Biétry et al. (2017)	The association between former BDZ use and the risk of AD. Data from the Helsana Group, a large Swiss health insurance provider.	2013–2014	1,438 AD patients and 1,438 controls.	Diagnosis of AD in 2013 or 2014 *via* recorded first-time use of acetylcholinesterase inhibitors or the N-methyl-D-aspartate receptor antagonist memantine (agents commonly used to treat AD) using anatomic therapeutic chemical classification (ATC) codes N06DA02 for donepezil, N06DA03 for rivastigmine, N06DA04 for galantamine, or N06DX01 for memantine	BZD use in the 2 years preceding dementia diagnosis was not associated with an increased risk of developing AD.
Saarelainen et al. (2018)	To investigate the risk of death associated with new BZD and related drug (BZDR) use in a nationwide cohort of persons with AD. MEDALZ cohort in Finland. (Finland)	2005–2011	70,718 AD patients.	AD diagnoses based on the National Institute of Neurologic and Communicative Disorders and Stroke and the Alzheimer's disease and Related Disorders Association as well as the Diagnostic and Statistical Manual, Fourth Edition criteria.	BZD use is associated with an increased risk of death in persons with AD.
Tapiainen et al. (2018)	To assess the association between BDZ and related drug use and risk of AD, considering cumulative consumption and duration of treatment.	2005–2011	70,719 subjects with clinically verified AD diagnosis in 2005–2011 and 282,862 matched controls.	AD diagnosis based on DSM-IV and NINCDS-ADRDA criteria. Several confounding factors were considered, such as chronic diseases (COPD, asthma, cerebrovascular dementia, diabetes), abuse of other substances, socioeconomic status and the use of antidepressants or antipsychotics 5 years before the diagnosis of AD.	BZD and related drug use was associated with a modestly increased risk of AD. No major differences were observed among different subcategories of BZDs (BZDs, Z drugs, short-/medium-acting or long-acting BZDs)

In a nested case–control study, Lagnaoui et al. concluded that there was a small increased risk of dementia after the administration of GABA_A_ activators (Lagnaoui et al., 2002). Notwithstanding, the authors acknowledged that the effect of exposure to other non-evaluated drugs with possible Central Nervous System (CNS) effects, such as antipsychotics, could have biased the results. In another study, the same group evaluated the prevalence of BZD use in AD patients for 3 months, raising awareness about the use of BZDs in elderly AD patients (Lagnaoui et al., 2002). The same authors conducted a case–control study with data from a large representative cohort of Canadian older women in order to examine the association between BZDs and AD. They found a non-significant tendency toward an association between former use of BZDs and increased risk of cognitive decline. Perhaps the low number of cases (*n* = 73) and controls (*n* = 437) prevented reaching statistical significance.

Wu et al. conducted two case–control studies in Taiwan using the National Health Insurance Research Database (NHIRD) with data of people aged ≥45 from 1997 to 2004 (Wu et al., 2009). The main conclusions of the study were that long-term use of BZDs or similar drugs might be associated with increased risk for dementia and cognitive alterations in prevalent and chronic users over a maximum follow-up of 8 years. The authors also suggested that risk of dementia was associated with higher cumulative dosage and longer duration of BZD exposure (Wu et al., 2009). In the second study, the same group stated that this association was reversible since BZD discontinuation could decrease the risk of dementia (Wu et al., 2011).

In 2012, Billioti de Gage et al. performed a study in a French population. The main conclusion was that the new use of BZDs was associated with an about 50% increase in the risk of AD (Billioti de Gage et al., 2012). In 2014, the same group assessed the effects of exposure to BZDs among 10 up to 5 years before the diagnosis of AD, considering both the doses and the reason for prescription (anxiety, depression, insomnia) in a population of aged individuals of Quebec (Canada) (Billioti de Gage et al., 2014). They concluded that the chronic use of BZDs was associated with a higher risk of AD when daily doses ranged between 91 and 180 mg/kg (cumulative dose expressed as prescribed daily, during 3–6 months), and increased further with doses higher than 180 mg/kg (during more than 6 months of exposure).

Gomm et al. reported that the risk of dementia increased by 21% in patients receiving regular hypnotic drug prescriptions when compared to non-users (odds ratio [OR] 1.21, 95% confidence interval [CI] 1.13–1.29; *p* < 0.001) (Gomm et al., 2016). The authors also reported the existence of a period of about 3 years from the first prescription of the BZDs to the diagnosis of dementia. This study did not create specific selection methods for those individuals with higher potential risk of AD, such as those with APOE4 allele or low educational level. Likewise, the study only focused on the analysis of regular hypnotic users.

Saarelainen et al. conducted a study in a Finnish cohort of 70,718 subjects diagnosed with AD between 2005 and 2011 in order to investigate the effects of BZDs and Z-drugs in a population of AD patients compared with matched controls (Saarelainen et al., 2016). The authors concluded that these psychotropic drugs could inhibit the beneficial effects of the drugs used in the treatment of AD, either anticholinesterases or memantine. Furthermore, AD patients treated with BZDs showed a risk of mortality up to 41% higher than those who did not use such drugs. Z-drugs did not increase the risk of death, but authors suggested that they could not be considered safer in persons with dementia (Saarelainen et al., 2018).

Another study was performed in a wide population of Finland for 6 years (Tapiainen et al., 2018). Currently, this is the largest study assessing the effect of BZDs and Z-drugs on AD risk. Moreover, the authors concluded that BZDs and Z-drugs modestly increased AD risk since the Off Ratios (OR) after adjusting for another concomitant psychotropic medication was 1.06 (95% CI 1.04–1.08). They did not find significant differences between BZD subcategories (long or short action).

Controversially, there are some studies questioning the notion that BZDs/Z-drugs increase the risk of cognitive loss. For instance, in a study performed by Infeld et al., the results showed that long term use of BZDs did not increase the risk of AD (Imfeld et al., 2015). Adjusted odds ratios (aORs) were calculated with 95% confidence intervals (CIs) of developing AD or VaD in relation to previous BZD use, stratified by duration and benzodiazepine type. The OR of developing AD for those who started BZDs 1 year before AD diagnosis was 2.20 (1.91–2.53) and fell to the null for those who started between 2 and 3 years before [aOR 0.99 (0.84–1.17)] (Imfeld et al., 2015). In the same line, Biétry et al. evaluated the risk of AD after a period of 2 years of BZD and related drug treatment before AD diagnosis (Biétry et al., 2017). The results of the study indicated that the risk of developing AD was not associated with BZDs or Z-drugs. Likewise, the half-life of BZDs was not linked with AD risk (Biétry et al., 2017).

### Cohort Studies

Data from studies reviewed in this section can be found in Table 2.

**Table 2:** Cohort studies.

**References**	**Objective**	**Intervention**	**Number of subjects**	**Main outcome measures**	**Findings**
Lopez et al. (1999)	To examine the association of psychotropic medication use with cognitive, functional, and AD	1983–1988	179 patients with Alzheimer's disease age 82.2 mean 6.6	Cognitive impairment was evaluated using the Mini-Mental Status Evaluation (MMSE)	BZDs increase the risk of AD
Ellul et al. (2007)	To examine the effects of several drugs on the progression of disease in patients with Alzheimer's disease.	Not reported	257 patients with Alzheimer's disease age 82.2 mean 6.6 standard deviation	The diagnosis of Alzheimer's disease was made according to NINCDS-ADRDA criteria	Antipsychotics and BZDs were associated with a greater cognitive decline in patients treated with these drugs.
Rosenberg et al. (2012)	To examine the longitudinal association of psychotropic medication through the Persistency Index, which represents years of drug use divided by years of observation following AD diagnosis with cognitive, functional, and neuropsychiatric symptom among community-ascertained incident AD cases from the Cache County Dementia Progression Study	Not reported	335 participants were diagnosed with incident AD	Cognitive impairment was Mini-Mental State Evaluation (MMSE) and Clinical Dementia Rating	Psychotropic medication use was associated with more rapid cognitive and functional decline in AD
Hessmann et al. (2018)	To evaluate the continuity of BZDs prescriptions in patients with dementia insured in a German public sickness fund (Allgemeine Ortskrankenkasse AOK, 2018) in Lower Saxony, Germany	2014–2015	1,298 subjects with dementia.	Diagnosis of dementia in 2014, identified according to the International Classification of Diseases	The use of long acting BZD should be avoided in dementia patients.
Lee et al. (2018)	Association between sedative-hypnotic use and the risk of AD, in a Korean population through a retrospective cohort study from the National Health Insurance of Korea database	2002–2015 follow-up.	268,170 subjects. Age ≥ 50 The dosage of sedative-hypnotics was standardized by defined daily dose (DDD).	Comparison between the ever exposed, who were prescribed over 30 DDD of sedative-hypnotics and the non-exposed.	The risk of AD was higher in subjects exposed to sedative-hypnotics. (GABAA receptor agonists). Patients exposed to over 360 DDD of sedative-hypnotics showed a higher risk of dementia when compared to non-treated patients
Grande et al. (2019)	To investigate the effect of BDZs on first cognitive alterations in primary care patients suffering early cognitive alterations. Data comes from the REMIND—REteMilanese INtegrata per le Demenze— database.	Not reported	4,249 subjects (mean age 77.0 ± 8.2) enrolled by 353 General Practitioners (GPs) in the Milan metropolitan area.)	Evaluation of cognitive function by *ad hoc* trained GPs, using the Mini Mental State Examination (MMSE).	BZD use is not associated with an increased risk of poorer cognitive performance in primary care patients with first cognitive complaints.

Several prospective and retrospective cohort studies which have assessed the association of BZDs/Z-drugs and related drugs on cognitive function reported controversial results.

For example, the study of Lopez et al. concluded that the use of BZDs in AD patients should be done with great caution and its use would not be adequate due to the risks of falls (Lopez et al., 1999). Also, in the treatment of insomnia, they suggested the use of other medications such as antihistaminic drugs. Also, Ellul et al. suggested that the prescription of antipsychotics and BZDs can accelerate cognitive decline in patients with AD (Ellul et al., 2007). In the prospective long-term “Caerphilly study,” results also evidenced the association between the use of BZDs and the increased risk of developing both vascular and non-vascular dementia (Gallacher et al., 2012). The authors studied a representative sample of men with a follow-up rate over 22 years. They reported a significant higher risk for dementia with the use of BZDs. Moreover, Rosenberg et al. suggested that antipsychotics and BZDs showed an increase in cognitive loss associated with a high Persistency Index (Rosenberg et al., 2012). Lee et al. evaluated the risk of AD after the use of sedative-hypnotic antidepressants and antipsychotic drugs (Lee et al., 2018). Interestingly, the risk of AD was higher in patients receiving a defined daily dose over 30 defined daily dose (DDD). Likewise, in this study, different groups of BZDs were evaluated, and intermediate BZDs were associated with the highest risk of dementia. This study concluded that the risk of AD was associated with BZDs and sedative-hypnotic drugs, and that this association was dose-dependent (Lee et al., 2018). In another study performed in Taiwan, Chen et al. used data from the NHIRD, which covered 23 million registered patients from 1995 to 2010, accounting for 99% of the population (Chen et al., 2012). The authors clearly indicated the hypnotic drugs used which were classified into two groups: BZDs and Z-drugs. The results suggested that both hypnotics should be considered risk factors for dementia in patients with long-term insomnia. Likewise, an association between higher prescribed dosages of BZDs and Z-drugs and risks of dementia was found, which is in agreement with previous studies (Tapiainen et al., 2018).

It has been proposed that older people with anxiety may have a higher risk of AD development and involved an increase of Aβ levels in adults with MCI and AD (Pietrzak et al., 2015). Thus, Burke et al. investigated the role of anxiolytic drugs in the risk of AD. Likewise, they evaluated the association of APOE ε4, currently the most important risk factor in LOAD (Burke et al., 2018). One important finding of the study was that ε4 carriers had a statistically higher significant risk of AD development; however, this effect was moderated by the use of anxiolytics. Anxiolytics, alprazolam, lorazepam, paroxetine, or venlafaxine, specifically, may improve the association of anxiety on MCI and AD development. However, in the same study the authors reported that clonazepam conferred a statistically significant increased risk of MCI development among users of ε4 with anxiety, suggesting that there is a molecular mechanism on cognition that is altered by clonazepam.

Divergently, the prospective population-based cohort study published by Gray et al. investigated the risk of dementia associated with cumulative dosage of BZDs after an average follow-up of 7 years (Gray et al., 2016). They reported a small increased risk of dementia in people with low or moderate BZD treatment. However, the study concluded that BZDs do not increase the risk of AD (Gray et al., 2016). Furthermore, another team examined patients with recently diagnosed mild AD which were treated with anti-dementia medications such as acetylcholinesterase inhibitors (donepezil, galantamine, rivastigmine) and memantine. The authors reported an association between AD and an increased use of psychotropic drugs (Törmälehto et al., 2017). However, the administration of psychotropic medications was not related to alterations in cognitive performance (Törmälehto et al., 2017). Finally, Grande and collaborators showed that patients treated with short- and long-acting BZDs presented adjusted MMSE mean scores of 25.4; 95% CI 25.1–25.7, while non-treated patients had 25.9; 95% CI 25.3–26.4 (short acting BZDs); 25.3; 95% CI 25.2–25.5 (long-acting BZDs); (*p* = 0.156) (Grande et al., 2019). Therefore, the authors stated that the use of BZDs was not associated with an increase in cognitive loss in patients suffering initial cognitive alterations.

### Longitudinal Studies

Data from studies reviewed in this section can be found in Table 3.

**Table 3:** Longitudinal studies.

**References**	**Objective**	**Intervention**	**Number of subjects**	**Main outcome measures**	**Findings**
Bierman et al. (2007)	To evaluate the effects of BZD use on cognitive function in the elderly. Data from the Longitudinal Aging Study Amsterdam (LASA), a population-based study	9 year follow-up	2,105 subjects aged 55 to 85 years.	General cognitive functioning measured by means of the Mini-mental State; Episodic memory measured with a Auditory Verbal Learning Test; Fluid intelligence measured by means of two sub-sets of 12 items (A and B) from Raven's Colored Progressive Matrices; Information processing speed measured by means of an adjusted version of the Coding task.	The duration of treatment and cumulative exposure to BZD use had a negative effect on the cognitive function of elderly people. However, this effect was small.
Boeuf-Cazou et al. (2011)	To investigate the impact of long-term BZD consumption on cognitive function Population from the VISAT study (Aging, Health and Work) (France). A prospective cohort study.	10 year follow-up	1,660 men and 1,577 women aged 32, 42, 52, and 62 years, classified according to the use of BDZs into non-users, occasional users and log-term users.	Cognitive function was assessed using five cognitive tests (immediate free recall test, delayed free recall test, recognition test, Digit Symbol Substitution Subtest and visual search speed test).	Long-term use of BDZs leads to specific impairment in long-term memory in women.

In a study performed in a young adult population, Boeuf-Cazou et al. concluded that, although long-term exposure to BZDs leads to specific impairment in long-term memory only in women, a longer period of observation is necessary to ascertain if these alterations are associated with a risk of developing dementia in old age (Boeuf-Cazou et al., 2011).

Yet, in a large population-based cohort study named “*The Three-City Study*,” Shash et al. compared short- vs. long-half-life BZDs as well as the effects of other psychotropic medications on dementia on non-institutionalized individuals aged ≥65 starting in 1999. The authors concluded that users of long half-life BZDs had a 60% increased risk of developing dementia (Shash et al., 2016). Also, it was examined whether the chronic use of BZDs over 4 years was associated with an increased risk of cognitive decline (Paterniti et al., 2002). The study demonstrated that prolonged use of BZDs was a significant risk factor for the cognitive decline in the elderly which was evaluated by the MMSE, the Trail Making Test, and the Digit Symbol Substitution. Finally, it had been described that BZDs decrease cognitive performance, although the effects were small. In addition, they suggested that higher BZD treatment duration and cumulative doses were responsible for the negative effects on cognition in elderly patients (Bierman et al., 2007).

### Cross-Sectional Studies

Data from studies reviewed in this section can be found in Table 4.

**Table 4:** Cross-sectional studies.

**References**	**Objective**	**Intervention**	**Number of subjects**	**Main outcome measures**	**Findings**
Taipale et al. (2015)	To investigate the prevalence of BZD and related-drug consumption, especially those of long-term, and its associated factors among community dwelling individuals with and without AD.	2002–2006	The number of persons included in the study was 24,966 for individuals with AD and 24,985 for individuals without AD. The research was based on data from the MEDALZ-2005 (Medication use and Alzheimer's disease) cohort, which includes all community-dwelling individuals, diagnosed with AD in Finland at the end of 2005 and matched individuals without AD.	The diagnosis of AD based on the INCDS-ADRDA and DSM-IV criteria.	The long-term use of BDZs may impair cognition and may be associated with serious adverse events.
Hessmann et al., 2019	To evaluate the continuity of BZD prescriptions among dementia patients in Lower Saxony, Germany.	2014–2015	98 subjects with dementia.	Diagnosis of dementia in 2014, identified according to the International Classification of Diseases	The use of long acting BZD should be avoided by dementia patients.

Mura et al. conducted a cross-sectional and longitudinal study to evaluate the effects of chronic BZD use on cognitive decline in people over 65 years old in France (Mura et al., 2013). A total of 5,195 persons were included in the study, 969 of which were chronic users of BZDZs. The results showed that chronic BZD use was associated with poorer cognitive performance, but not with accelerated cognitive decline with age. However, the authors stated that BZDs could deteriorate cognitive performance, increase the depletion of cognitive reserves and precipitate the onset of dementia (Mura et al., 2013). Later, another team aimed to evaluate the prevalence of BZD and Z-drug use in a population of Finland (Taipale et al., 2015). The authors concluded that approximately half of the people with AD used BZDs and Z-drugs during the 4 years of follow-up, and that AD patients used more BZDs in the long term that those without AD. In another study, the same group investigated the risk of any stroke (ischemic or hemorrhagic) associated with BZDs and Z-drugs in patients with AD taken from the MEDALZ-2005 population. They found an increase of 20% in the risk of ischemic stroke in AD patients using BZDs and Z-drugs. However, the risk of hemorrhagic stroke was not increased (Tolppanen et al., 2017).

Contrarily, Zhang et al. who collected data from the National Institute on Aging (NIA) ADCs, reported no association between BZDs and cognitive decline (Zhang et al., 2016).

### Meta-Analyses

Zhong et al. investigated the association of long-term BZD use and dementia in a meta-analysis including five studies, which involved 45,391 participants and 1,891 dementia cases. In addition, the authors evaluated the potential risk of dementia associated with an increase of BZD dose by 22% (risk ratio 1:22, 95% CI 1.18–1.25). They concluded that long-term BZD users had an increased risk of dementia compared with non-users (Zhong et al., 2015).

In turn, He et al. pooled 10 studies to assess the association between BZDs and risk of dementia. PubMed and Embase databases were systematically searched for relevant publications up to September 2017. The literature search focused on observational studies that analyzed the relationship between the long-term use of BDZs and the risk of dementia. Their main findings pointed to a significantly increased risk of dementia in the elderly population using BZDs. This effect was associated with the use of BZDs with a longer half-life and with a longer treatment duration (He et al., 2019).

Finally, a meta-analysis of 12 prospective and retrospective cohort studies and case–control studies about the risk of BZDs and AD was reported. The team concluded that the use of BZDs—mainly those of long-action—and Z-drugs was associated with the development of dementia. However, the study showed some limitations since it did not differentiate between BZDs effects on AD and vascular dementias, neither between long- and short-acting BZDs (Lucchetta, 2018).

## Discussion

In the present review, we discussed the evidence pointing to BZDs as risks factors for cognitive decline in aging and AD (Paterniti et al., 2002; Zhang et al., 2016; Picton and Pharm, 2018).

Sleep disorders increase with age, altering clearance of toxic and waste products from the brain, including Aβ (Clinton et al., 2011). It has been reported that sleep disturbances could contribute to neurodegeneration and AD by altering physiological metabolic functions, increasing oxidative stress, and the accumulation of Aβ as well as due to the appearance of neuroinflammatory responses (Phan and Malkani, 2019) (Figure 1). In addition, insomnia is also associated with hypertension, diabetes, and a higher obesity risk; all of them contributing to AD (Clarke et al., 2018; Frozza et al., 2018). Bearing this in mind and considering the increase in insomnia diagnostics, especially in the elderly, this situation has led to higher consumption of hypnotic drugs in the adult population to improve sleep quality. Notwithstanding, and seeing the possible consequences derived from their intake, there needs to be a significant increase in awareness about their risks. In fact, there have been some studies reporting that the consumption of BZDs favors the appearance of cognitive affectations and increases the number of deaths in patients with AD (Imfeld et al., 2015; Saarelainen et al., 2017; Grande et al., 2019). Yet, as we have already assessed, there is controversy, and other researchers defend that BDZs have no such detrimental effects (Picton and Pharm, 2018). Thus, even though BZDs improve various measures of insomnia in the aged population, their clinical value is debatable, and melatonin agonists could be a much safer choice when trying to manage sleep disorders (Investigations, 2015). For the treatment of anxiety in the elderly, the administration of other medications such as serotonin uptake inhibitors (sertraline) or other compounds could be more adequate (Consensus and Statements, 2014; Investigations, 2015).

In the end, the mechanisms by which BZDs and Z-drugs could increase the risk of cognitive loss and AD remain to be clarified; here we have discussed some specific hypotheses. Thanks to molecular biology, the α1 subunit of the GABA has been shown to play an important role in BZD-mediated cognitive loss. Hence, it has been reported that higher activity at the α1GABA_A_ receptors induced by positive allosteric modulation at the BZD site is responsible for spatial learning and memory incapacitation in preclinical models. Besides, activation of the α5 subunit, which is mainly expressed in the hippocampus, could in part explain the memory deficit states induced by BZDs. Therefore, α5GABA-targeting compounds could improve cognition, thus having therapeutic potential in AD and other dementias (Adrienn et al., 2017). It is conceivable that BZDs influence cognition and probably increase the risk of AD acting through hippocampal α5GABA_A_, while Z-drugs (α5GABA_A_-independent) confer a lower risk. In addition, the neuroinflammatory process is *per se* a risk factor for AD. The brain microglia play a prominent role in neuroinflammation, and it is associated with the secretion of pro-inflammatory cytokines. Likewise, α5GABA_A_ receptor activity is enhanced by the inflammatory process, a fact that is probably critical in inflammation-induced memory deficits (Marczynski, 1998). Moreover, BZDs and other drugs may increase cognitive loss and AD risk through the phosphorylation of tau protein, which can also inhibit the signaling of the brain insulin receptor (Jovanovic, 2004; Whittington et al., 2019).

In spite of these described effects, it could be hypothesized that BZDs may indirectly exert a protective effect against the development of AD by improving sleep (through their clinical effects on sleep latency, number of awakenings, and duration and quality of sleep) (Guzmán et al., 2018). This paradigm states that the enhancement of GABA_A_ receptor activity by BZDs could inhibit glutamatergic neurotransmission, thereby protecting against the excitotoxic effects of glutamate on the pathogenesis of AD (Fastbom et al., 1998). Moreover, it is noteworthy that some clinical trials reported no association between BZDs and risk of cognitive loss and AD.

In conclusion, we do not have enough data to ensure a causal relation between psychotropic drugs and cognitive loss. However, a therapeutic strategy based on BZDs and Z-drugs in elderly people should be extensively evaluated and monitored (Monzani et al., 2015; Yi et al., 2018). After reviewing the available data, a controversial question remains: is it safe to prescribe BZDs and Z-drugs to improve sleep in older patients, despite the potential cognitive loss risk? We strongly believe that there are enough data supporting an extremely cautious attitude with Z-drugs and the avoidance of BZD prescription in elderly people with AD.

## Author Contributions

All the co-authors of this research (ME, ACan, OB, PM, RC-T, CB-Z, MG, JO, CA, JF, and ACam) have directly participated in the planning, execution of the manuscript. All authors have read and approved the final version submitted.

### Conflict of Interest

The authors declare that the research was conducted in the absence of any commercial or financial relationships that could be construed as a potential conflict of interest.
